# Vaccination status, awareness, and its correlates among healthcare workers in the Delhi-National Capital Region (NCR): a mixed-method study

**DOI:** 10.3205/dgkh000494

**Published:** 2024-08-20

**Authors:** Mohammed Ahmed, Varalakshmi Manchana

**Affiliations:** 1 School of Medical Sciences, University of Hyderabad, Gachibowli, Hyderabad, India

**Keywords:** vaccination, vaccination status, healthcare workers, infection control, mixed-method

## Abstract

**Introduction:**

Healthcare workers (HCWs) are on the frontline of infections. To safeguard HCWs from occupational exposure to infections and to curb nosocomial infection a set of vaccines has been recommended for them by the WHO. Hence, we aimed to assess the vaccination status, awareness, and its correlates amongst HCWs in the Delhi-NCR.

**Method:**

The study used a cross-sectional mixed-method approach from January to April 2023. For the quantitative arm, a structured questionnaire was circulated to the participants in conveniently-selected private and government tertiary care hospitals of the Delhi-NCR, both through e-survey using Google form, and in person, data were collected on socio-demographics, vaccination status, and awareness. SPSS version 25 was used for the analysis. For the qualitative arm, in-depth interviews were conducted and data were analyzed manually.

**Results:**

Out of 387 participants (62.8% males, 37.2% females), the awareness about the vaccines recommended for HCWs was 64.1%. However, only 15.3% of HCWs were completely aware of all the recommended vaccines. SARS CoV-2, Polio, Hepatitis B, and BCG had the highest vaccination coverage, 97.4%, 87.9%, 83.7%, and 50.9%, respectively. It was found that gender, education, type (private or governmental) of tertiary care hospital, and profession had a significance (p<0.05) on the vaccination status score and awareness of all WHO-recommended vaccines (AOR=7.6, 95% CI, 3.24–18.0). The qualitative arm further augmented the findings.

**Conclusion:**

The study reveals insufficient awareness and vaccination status regarding recommended vaccines. Prioritizing the preparation of unified standard guidelines for Indian HCWs and involving concerned stakeholders is crucial.

## Zusammenfassung

**Einleitung:**

Beschäftigte im Gesundheitswesen sind an vorderster Front mit Infektionen konfrontiert. Um sie vor berufsbedingten Infektionen zu schützen und nosokomiale Infektionen einzudämmen, empfiehlt die WHO eine Reihe von Impfungen für Beschäftigte im Gesundheitswesen. Daher sollten der Impfstatus, das Bewusstsein und Einflussfaktoren auf die Impfbereitschaft bei Beschäftigten des Gesundheitswesens in der Delhi-National Capital Region (NCR) untersucht werden.

**Methode:**

Für die von Januar bis April 2023 durchgeführte Studie wurde ein Querschnittsansatz mit gemischten Methoden verwendet. Für den quantitativen Teil der Studie wurde ein strukturierter Fragebogen über eine Online-Plattform an die Teilnehmer in ausgewählten privaten und staatlichen Tertiärkliniken in Delhi-NCR verschickt, und es wurden Daten zu soziodemografischen Daten, Impfstatus und Bewusstsein erhoben. Für die Analyse wurde SPSS Version 25 verwendet. Für den qualitativen Arm wurden Tiefeninterviews durchgeführt und die Daten manuell ausgewertet.

**Ergebnisse:**

Von 387 Teilnehmern (62,8% männlich, 37,2% weiblich) waren 64,1% über die für das Gesundheitspersonal empfohlenen Impfstoffe informiert. Allerdings waren nur 15,3% vollständig informiert. Bei SARS CoV-2, Poliomyelitis, Hepatitis B und BCG war die Durchimpfungsrate mit 97,4%, 87,9%, 83,7% bzw. 50,9% am höchsten. Geschlecht, Bildung und der Typ des Krankenhauses der Tertiärversorgung (privat oder öffentlich) hatten einen signifikanten Einfluss (p<0,05) auf den Impfstatus und die Aufklärung. Der qualitative Arm ergänzte die Ergebnisse.

**Schlussfolgerung:**

Die Studie ergab, dass der Bekanntheitsgrad der empfohlenen Impfstoffe und der Impfstatus unzureichend sind. Die Ausarbeitung einheitlicher Standardrichtlinien für das indische Gesundheitspersonal und die Einbeziehung der betroffenen Interessengruppen ist von entscheidender Bedeutung.

## Introduction

According to the existing evidence, India is going through a major epidemiological transition that has led the causes of death to shift from communicable diseases to more non-communicable diseases [1]. Nevertheless, infectious diseases are still a threat to human health and global stability, as proven again by the COVID–19 pandemic. In reality, infectious diseases have increased in frequency and complexity over recent years, with noteworthy examples being the Middle East respiratory syndrome coronavirus, the severe acute respiratory syndrome (SARS), influenza A subtype H5N1, Zika, and Ebola [2]. Several interlaced factors, e.g., high-density urbanization, virus mutation, climate change, increased global connectivity, migration, intensification of animal and plant trade, and altered human behaviour are creating opportunities for infectious diseases to emerge, re-emerge and spread [3]. 

Healthcare workers (HCWs) are particularly at risk and are prone to healthcare-associated infection, which makes them a key demographic for vaccination [4]. Vaccinating healthcare personnel (HCP) is a crucial preventative strategy to safeguard them and their patients from communicable diseases. With 3.8 million HCP, the healthcare industry is one of the top employers in India; however, few of these individuals are aware of the country’s immunization recommendations. According to data from infectious disease outbreaks across India, HCP have insufficient vaccination rates. Studies have found that the rates for influenza and varicella vaccinations are as low as 4.4% and 16%, respectively [5].

In the past ten years, the healthcare infrastructure and services in the Delhi NCR have grown at an unparalleled rate. The Delhi NCR is one of the most popular healthcare destinations in India for foreign patients due to its strong medical infrastructure, cutting-edge medical specialties, affordable costs, and highly qualified specialists. Moreover, It’s well known that urbanization, high population density, global connectivity, high volume of tourism, and population mobility contribute to the proliferation of infectious diseases [6], [7], thus vaccination remains the cornerstone of protection for HCP and their patients. For this reason, healthcare professionals should be aware of and vaccinated according to the recommended immunization schedule. Hence, the objectives of the present study were:

1. to assess the awareness of the WHO’s recommended vaccines;
2. to assess the vaccination status among the health care workers;
3. to assess the significance of socio-demographic variables on composite vaccination status and determine the socio-demographic factors associated with awareness of all the WHO-recommended vaccines (complete awareness);
4. to explore the barriers to and facilitators of vaccination status and awareness.

## Materials and methods

### Study design and setting

An embedded mixed-method cross-sectional study was conducted from January to April 2023 in private and government tertiary care hospitals of the Delhi-NCR. 

### Study participants

Study participants were HCWs, which includes physicians, nurses, medical technicians, students and trainees, pharmacists, general duty assistants (GDA), and people not directly involved in patient care but potentially exposed to infectious agents (housekeepers, security guards, maintenance, administrative and billing officials). 

### Sample size determination and sampling technique

Software version 3.0 of OpenEpi was used to determine the sample size. As there was no relevant literature, the percentage of HCWs who received the WHO-recommended vaccines was presumed to be 50%. With a 95% confidence interval and an absolute precision of 5%, a sample size of 384 was calculated. After accounting for a 10% nonresponse rate, the final sample size was 422. A convenience sampling technique was used to select the private and government tertiary care hospitals of the Delhi-NCR.

### Inclusion and exclusion criteria

The study only included HCWs without contraindications to the WHO-recommended vaccines. HCWs were excluded if they had contraindications or refused participation, and study did not inquire about booster doses. Furthermore, cholera and ebola vaccines were not included, as they are only recommended in appropriate high-incidence settings, such as emergencies.

### Data collection procedure

The quantitative data were collected through the E-survey using Google forms and in-person mode. Conveniently selected hospitals were physically visited by the principal investigator (me) and subsequently consent was sought for the study from the hospital administration and then with the help of questionnaire/in-depth interview guide data was collected. While collecting the data, inclusion and exclusion criteria was intact.The content of the questionnaire was validated by two experts. The feasibility and validity of the questionnaire were evaluated by a pilot study with 10 participants, and the feedback was simultaneously integrated. Sociodemographic characteristics of the participants included gender, age, education, type of tertiary care hospital, profession, and work experience. Awareness about the recommended vaccines was determined by asking the participants whether the WHO has recommended the vaccines for HCWs or not; if they responded with “yes”, then they were asked about the names of the recommended vaccines to differentiate partial and complete awareness. With regard to vaccination status, participants were asked about having been vaccinated, whether they had received the complete dosage, and documented proof of the vaccination, and simultaneously, the responses were recorded as “yes” or “no” or “don’t remember”. For qualitative data, a semi-structured in-depth interview guide was used. Certain participants among the research subjects were purposively selected for the face-to-face in-depth interviews until the theoretical saturation was reached. 

### Data processing and analysis

A total of 387 participants were eventually taken into consideration for the study, after the data obtained via the Google form and in-person mode were converted to an MS-Excel format and reviewed for completeness, consistency, and consent. For statistical analysis, data was exported to SPSS version 25 software. The exported data was categorized and summarized using descriptive statistics. To assess the significance of socio-demographic variables on vaccination status, each participant’s composite vaccination score was determined by allocating a value to their responses on their vaccination status, dosage completion, and supporting documentation, which was subsequently converted into a percentage. For continuous data, non-parametric tests were conducted for the comparison of differences. If there were two groups, we used the Mann-Whitney U-test. In case of more than two groups, the Kruskal-Wallis test was employed. For categorical data, the chi-squared test was used to determine statistical significance, and logistic regression was performed to find the crude odds ratio and adjusted odds ratio (CI 95%). A p-value <0.05 was considered statistically significant. The interviews were first transcribed before being coded and thematically analysed. 

### Ethical consideration

Ethical approval was obtained from the Institutional Ethics Committee (IEC), University of Hyderabad (UH/IEC/2022/395). 

## Results

### Quantitative findings

#### Socio-demographic characteristics of participants

Among the 387 participants, 243 (62.8%) were males and 144 (37.2) were females. Half of the HCWs (49.9%) were in the age group of <25 years, and the mean age of the HCWs was 27.5 (median=26, IQR 7) years (Table 1 (Tab. 1)).

#### Awareness of WHO recommended vaccines among HCWs

**Figure 1:**
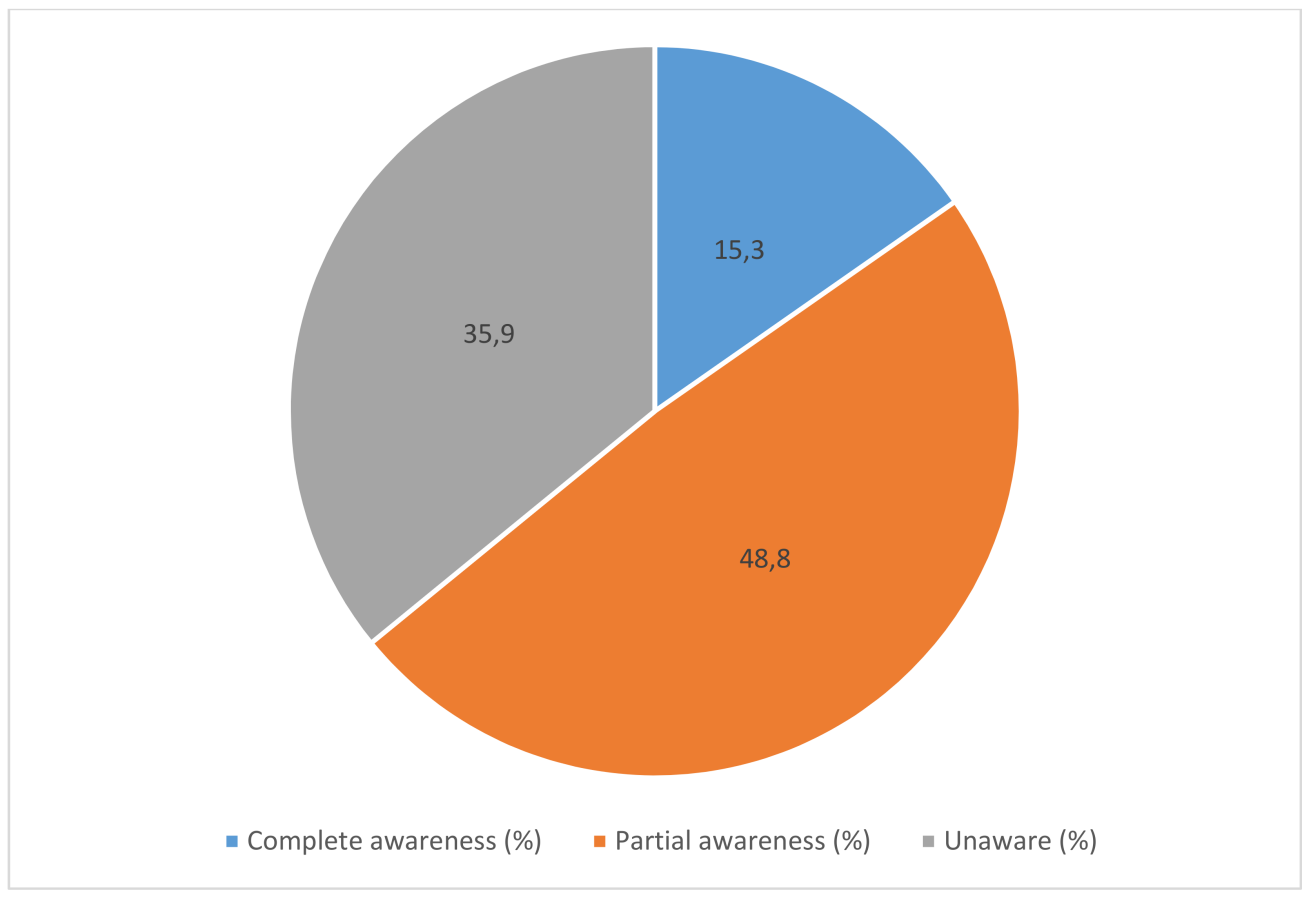
Awareness about the recommended vaccines among the HCWs

The awareness about the WHO’s recommended vaccines for HCWs was 64.1% (248). However, out of 248 only 59 (15.3%) of HCWs were completely aware of the recommended vaccines (Figure 1 (Fig. 1)). 

#### Vaccination status of HCWs with the WHO-recommended vaccines

**Figure 2:**
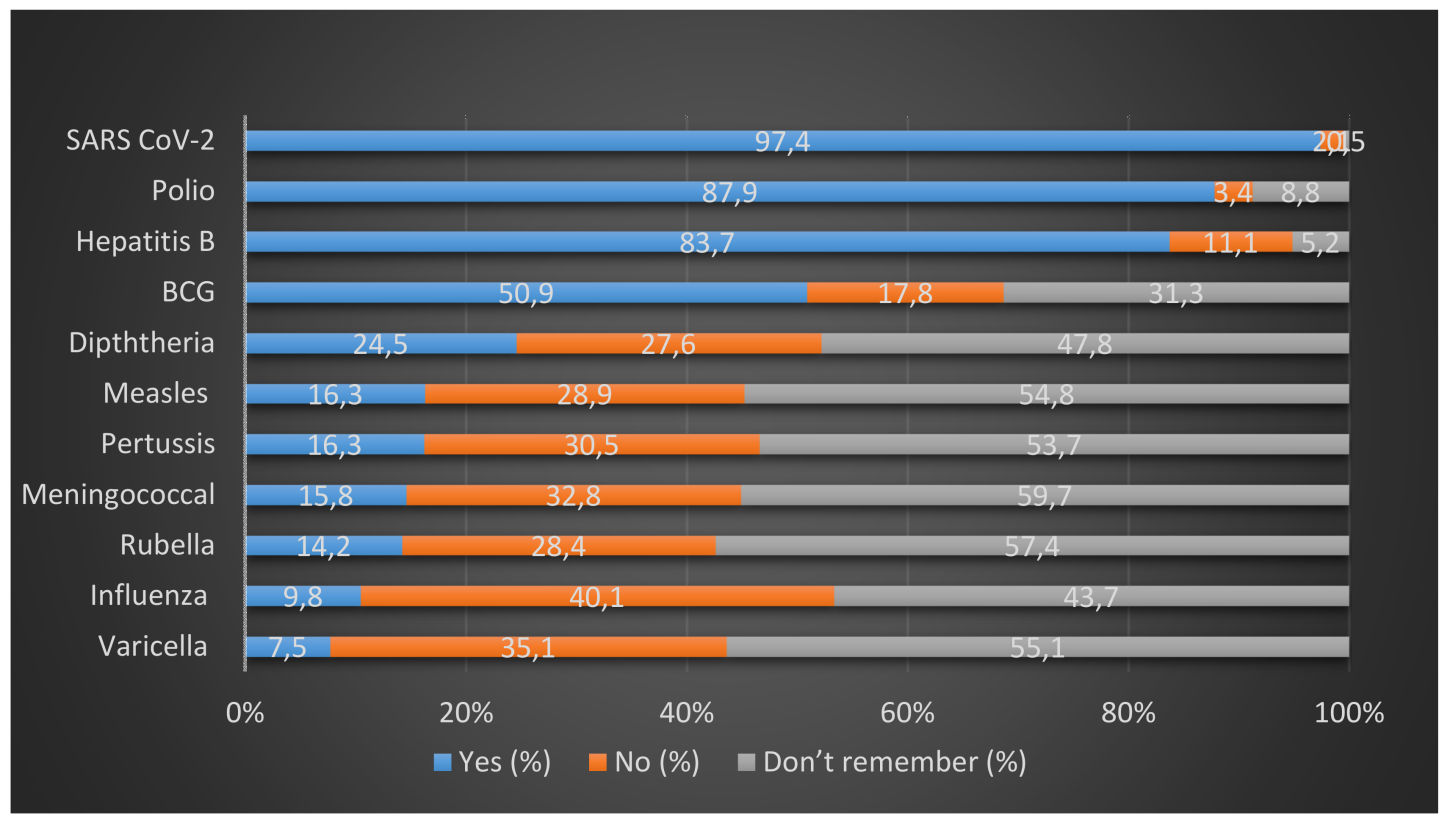
Vaccination status of HCWs

SARS CoV-2, polio, hepatitis B, and BCG had the highest vaccination coverage, i.e., 97.4%, 87.9%, 83.7%, and 50.9%, respectively. Diphtheria vaccination coverage was 24.5%. A vaccination rate of <20% was recorded for the rest of the vaccines. Out of those vaccines with <20% coverage, except influenza, more than 50% of the population did not remember whether they had been vaccinated or not (Figure 2 (Fig. 2)).

**Figure 3:**
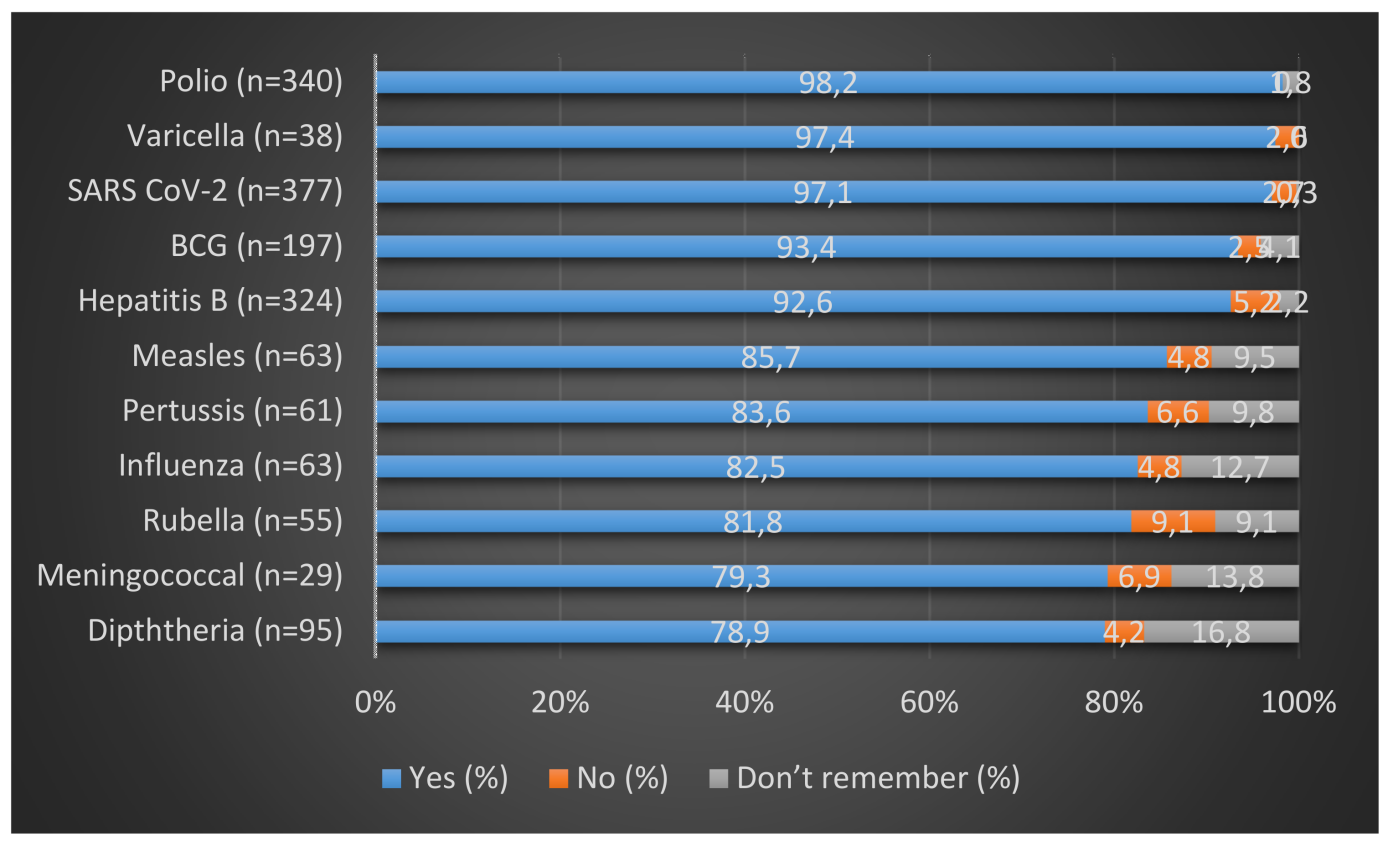
Vaccination dosage-completion status of HCWs out of those vaccinated

Over 90% of the individuals who received the BCG, SARS CoV-2, hepatitis B, polio, and varicella vaccines took complete doses, while for pertussis, measles, rubella, and influenza, an over 80% complete dosage coverage was reported. Notably, the vaccines pertussis (15.8%), measles (16.3%), rubella (14.2%), varicella (9.8%) and influenza (16.3%), for which a <20% of vaccination coverage was reported, demonstrated complete dosage coverage in >80% of those vaccinated (Figure 3 (Fig. 3)). 

Among those who were vaccinated against SARS-CoV-2, Hepatitis B and BCG, 91%, 61.1%, and 41.1%, respectively, had documented proof of vaccination. Documented proof of vaccination with the rest of the vaccines ranged from 13.2% to 35.5%. 

#### The significance of socio-demographic factors on composite vaccination status

Gender, education, type of tertiary care hospital and profession had a significant impact (p<0.005) on vaccination status. In terms of gender, females had a significantly higher median vaccination status score, 29.4%, compared to males with 26.4%. Regarding education, postgraduates (>5 years of tertiary education) had the highest median score of 33.82, followed by 29.41 for those with a diploma (1–3 years of tertiary education). The lowest score was noted for HCWs without formal education, with a median score of only 17.65. Government hospitals had a significantly higher median score of 35 compared to private hospitals, where the median score was 26. In relation to profession, doctors performed much better than the rest of the group, with a median score of about 53, followed by by nurses with 35 and pharmacists with a median score of about 28. The rest of the professions ranged between 17.6 and 26.4 (Table 2 (Tab. 2)). 

#### Factors associated with complete awareness

Education, type of tertiary care hospital and profession had a significance on awareness (chi-squared test, p<0.05). However, multivariate analysis of factors associated with complete awareness revealed that only the type of tertiary care hospital was statistically significantly associated with complete awareness. HCWs who work in government tertiary care hospitals were 7.6 times (AOR=7.65, CI 95%: 3.249–18.016) more likely to have complete awareness than to those who work in private tertiary care hospitals (Table 3 (Tab. 3)). 

### Qualitative findings

During qualitative data collection, a total of nine in-depth interviews were conducted to explore the barriers to and facilitators of vaccination status and awareness. The qualitative data were summarized in 7 themes by the use of thematic analysis. Under the themes, multiple codes were identified and supported by the related quotes. The themes included were:

- Self-perceived risk of infections at health care settings – participants perceived high infection risk due to inappropriate control measures, high disease prevalence, and broad transmission spectrum. Self-perceived risk influences vaccination rate, as our participants expressed willingness to be vaccinated.
- Perception about vaccine’s effectiveness and efficiency – it was observed that participants’ trust in vaccine effectiveness and motivation to be vaccinated, especially against prevalent infectious diseases, is influenced by perception and family involvement in healthcare.
- Concerns related to vaccination – participants were aware of side effects and complications of vaccines, but they considered them minor inconveniences. It was seen medical practice addresses phobia.
- Additional personal factors affecting the vaccination – personal factors such as un-mindfulness, previous experience, lack of time, and phobia affect vaccination confidence among HCWs.
- Vaccine affordability and accessibility – participants discussed vaccine affordability, particularly HPV vaccine. Although HPV vaccine is not on the WHO list of vaccines recommended for HCWs, it was used as an example by the participants to talk about vaccine affordability. The majority expressed concerns about financial backing by the government for health care workers; the non-availability of newer vaccines was also discussed.
- Role of officials and official bodies in promoting vaccination – the theme emphasizes enforcement, communication, official circulars, strictness, and freedom of choice for HCWs, with some supporting stricter recruitment and some expressing concerns about professional impact on their (HCWs) profession.
- Additional system-related factors affecting vaccination acceptance – participants advocated sick leave to recover from side effects. They recognized the role of the media in promoting accurate information, and suggested improved vaccination awareness and convenience (Attachment 1).

## Discussion

To the best of our knowledge, this is the first mixed-method study to determine vaccination coverage, awareness, and its correlates in terms of the WHO-recommended vaccines in HCWs of the Delhi-NCR. Our study found that 64.1% of the HCWs knew that there are vaccines recommended by the WHO for HCWs, although only 23.8% of them knew the names of the all recommended vaccines. The lack of consistent standard recommendations for vaccination among HCWs in India has been identified as the reason for the low level of recommended-vaccine awareness among this group, according to a recent narrative review [5].

In terms of vaccination status of HCWs, highest vaccine vaccination coverage rates were for SARS-CoV-2, polio, hepatitis B, and BCG, with 97.4%, 87.9%, 83.7%, and 50.9%, respectively. High coverage of COVID-19 and polio vaccination can be attributed to the recent outbreak of COVID-19 and the decades-long pulse-polio immunization (PPI) campaign [6]. In our study, vaccination coverage of hepatitis B was similar to that of a study conducted among oral health care personnel in Mysore, India, which showed that 85.4% of the HCWs were vaccinated [7]. One of the reasons for a high hepatitis B vaccination rate in the Indian healthcare setting is that India has 40 million HBV carriers and 10% to 15% of the world’s HBV population. Of these, 15% to 25% develop cirrhosis and other consequences that increase medical expenses and hasten mortality [8]. Moreover, Indian recommendations about hepatitis B vaccination are uniform [5], and it is usually emphasized in Indian healthcare settings for HCWs [9]. Half of the participants reported receiving the BCG vaccination. However, given our population’s median age, there’s a strong possibility of recall bias, as BCG vaccination has been scaled up in India since the mid-1900s [10]. 

The vaccination rate against pertussis, measles, meningococcal vaccine, rubella, and varicella was 15.8%, 16.3%, 7.5%, 14.2%, and 9.8%, respectively. Notably, over 50% of the population don’t remember whether they were vaccinated against these or not. In terms of diphtheria and influenza, over 40% of the population mentioned the same. Thus, it can be argued that HCWs are heedless of their vaccination status, which can be attributed to a lack of official checks while hiring HCWs and not having the recorded data of vaccination. Our qualitative finding supports this argument, as one healthcare worker mentioned “I was asked only about my Hep-B and covid vaccination, nothing else was asked.”

With regard to having documented proof, except for SARS CoV-2, hepatitis B and BCG, a low rate of vaccination documentation was reported for all the vaccines (13.2–35.5%). This can be explained by the fact that no central electronic database exists for the vaccination record of HCWs nor for the general population, and keeping the hard copies of childhood vaccination records is difficult. However, as per the WHO guidelines, HCWs should have documented proof of relevant vaccinations or else be subjected to revaccination [11]. Effective policy implementation in this regard will help further, as a statistically significant improvement was observed in keeping written vaccination records after the policy implementation [12]. A recently launched U-WIN app to record the vaccination status of newborns and pregnant women is also a hopeful sign in this regard [13].

In terms of being vaccinated with the complete dosage, it is notable that of the number of participants who were vaccinated, none of them reported less than 79% vaccine dosage completion. Our findings were similar to the study conducted in south India which found more than three-fourths of the participants (76.5%) received complete doses of HBV [14]. Hence, it can be argued that HCWs are well informed about the importance of being vaccinated with the complete dosage. However, this study does not allow a direct comparison with the study of Thazha et al. [14] because it did not check dosage compliance of other vaccines.

In our study, it was found that gender, education, type of tertiary care, and profession had a significance on vaccination status scores. In previous studies, males were seen to have better vaccination coverage [15], [16]. In contrast, our study reports better coverage among females, which may be because of our different dependent variable (composite vaccination status score). Considering educational level, postgraduates had the highest median score of 33.82, followed by diploma-holders at 26.47. The lowest score was noted on those without a formal education, with a median score of only 17.65. Despite the fact that a graduate degree requires more years of study, the median vaccination status score of these graduates was less than that of participants with a diploma, which shows a lack of exposure to information amongst the graduates. The exposure to information positively impacts knowledge and vaccination coverage [17]. Government HCWs had better vaccination status scores. The reason behind this might be that of HCWs prefer to be vaccinated at government institutions, and tend to prefer domestic (Indian) vaccines [18]. In relation to profession, doctors performed much better than the rest of the HCWs, with a median score of about 53, which agrees with the existing literature [19], [20], [21], [22]. The lowest score (17.65) was recorded for the students and interns/trainees, which may be attributed to unaffordability, as our qualitative findings suggest that affordability is a major concern for the interns, as they have to budget their daily expenses to be vaccinated. It should be borne in mind that our vaccination status score is a composite of three variables – vaccination status, receiving the complete dosage, and possession of documented proof for the same. Therefore, no direct comparison with other studies is possible. Furthermore, the results of this study cannot be generalized for rural settings, as it was conducted in an urban setting. 

With regard to awareness, our multivariate analysis shows that government HCWs were 7.6 times more likely to have complete awareness than private ones. The fact that government HCWs have been involved in routine vaccination programs and that routine immunization has been at the forefront of public health interventions – possibly one of the largest by the government sector – could help to explain the reason for this [23].

The fact that this study uses a mixed methodology and includes both qualitative and quantitative findings is one of its merits. Another strength constitutes the investigation of the presence of supporting documents confirming vaccination and dosage compliance of vaccines. Limitations include the self-reported data, non-random sampling technique, and possibility of recall bias. A higher representation of males, graduates, private HCWs, nurses, and early-care professionals are other shortcomings of this study.

## Conclusion

Complete awareness of the recommended vaccines was found to be remarkably low. The vaccination status for a few vaccines exhibited good coverage. However, with regard to the composite vaccination score of the HCWs, the overall situation is quite poor. It is crucial to consider the preparation of standard unified recommendations for Indian HCWs. If enforcement is lacking, then at the least a hospital official’s continuous supervision would lead to better awareness and coverage. At the same time affordability, availability, and the role of the media need to be acknowledged. Our foremost recommendation is to conduct awareness drives in healthcare settings and create a centralized electronic vaccination record for each healthcare worker, which will help keep track of coverage. While recruiting healthcare workers, their vaccination status must be checked and, as necessary, vaccination should be facilitated.

## Notes

### Aknowledgement

The authors are thankful to all the participants of the study, healthcare workers and hospitals who facilitated the data collection procedure.

### Authors’ ORCID

- Mohammed Ahmed: 0000-0001-6132-4891
- Varalakshmi Manchana: 0000-0001-8072-2414

### Funding

This research received no external funding.

### Data availability statement

The study’s data, which support its findings, are available upon request from the author but not publicly available due to potential privacy concerns.

### Declaration of generative AI and AI-assisted technologies in the writing process

During the preparation of this work, the authors used Quillbot to paraphrase some of the contents to improve language and readability, given the non-native English background of the authors. After using this tool, the authors reviewed and edited the content as needed and take full responsibility for the content of the publication.

### Competing interests

The authors declare no competing interest.

## Supplementary Material

Themes, codes, and concerned quotes of qualitative analysis

## Figures and Tables

**Table 1:**
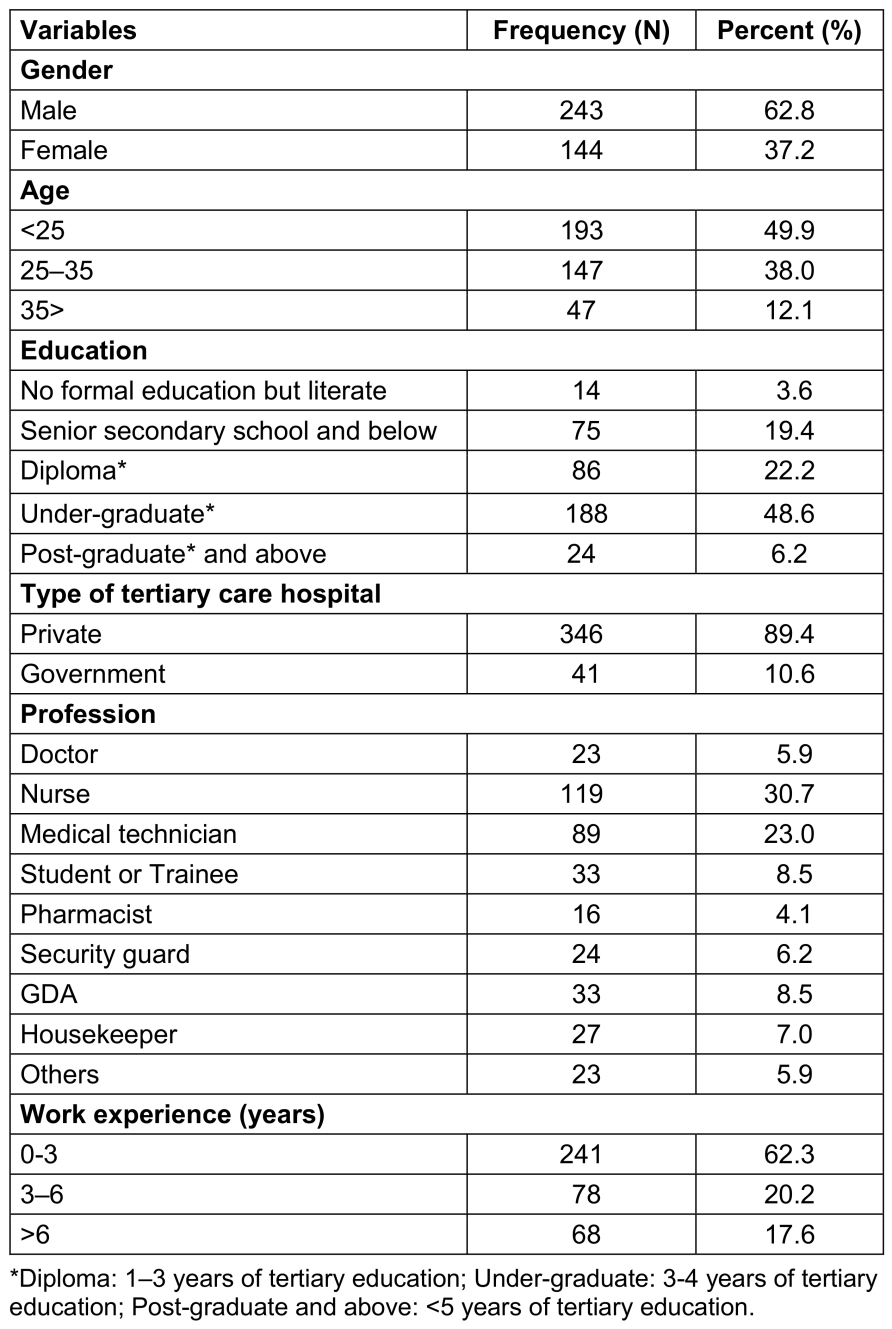
Socio-demographic characteristics of the study participants (N=387)

**Table 2:**
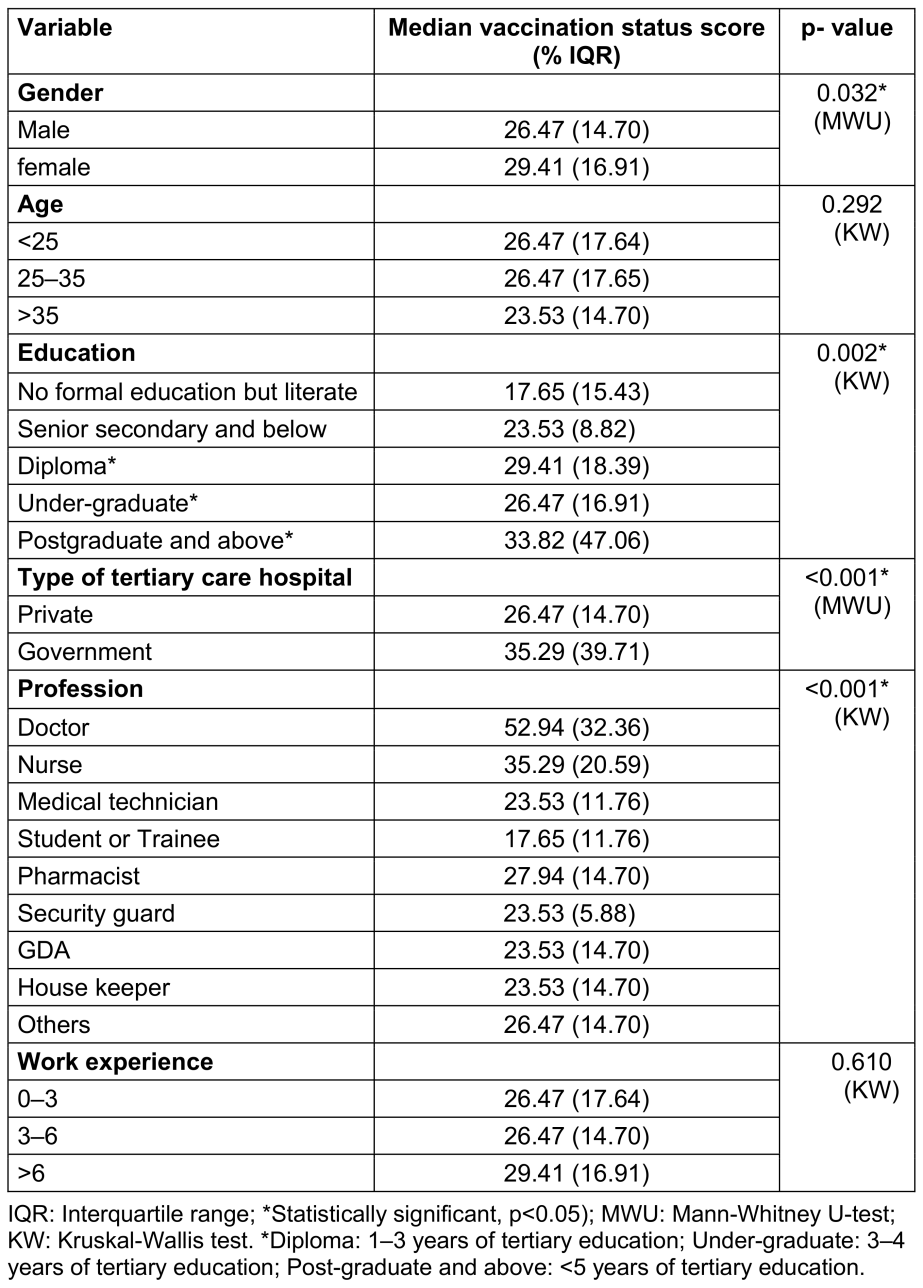
Comparison of vaccination status score according to different variables

**Table 3:**
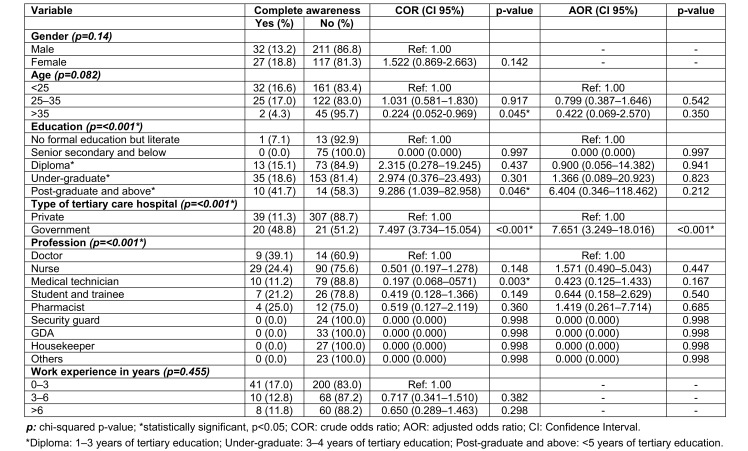
Logistic regression of factors associated with complete awareness of WHO-recommended vaccines
