# Supplementary material for: Vaccination status, awareness, and its correlates among healthcare workers in the Delhi-National Capital Region (NCR): a mixed-method study
Source: GMS Hyg Infect Control. 2024 Aug 20;19:Doc39. doi: 10.3205/dgkh000494 (PMC11367558; doi:10.3205/dgkh000494)
Supplement: Themes, codes, and concerned quotes of qualitative analysis [file HIC-19-39-s-001.pdf]

Attachment 1: Themes, codes, and concerned quotes of qualitative analysis

Themes	Codes	Unedited Quotes
**Self-perceived risk of infections in healthcare settings**	Inappropriate infection control measures in healthcare settings	*[….] "Yes, there's definitely healthcare, social infections, maybe due to hospitals not maintaining their standards." (1)*<br>*"Clothes and my belongings get contaminated while examining the patients, so there are chances." (09)*
	Not having personal safety measures as a risk factor	*[…] "If we don't maintain our personal hygiene and also if we don't sanitize ourselves […] it might be a risk of infection." (1)*<br>*"Also, if I'm doing any procedure without maintaining the proper sterility, then that patient might get infection from me too, right." (07)*
	Source of infection for others	*"I work in a hospital setting, I can Be a career if not, Infectious person myself, I could be a great career." (2)*<br>*"Yes, I fully consider myself as a source of infection for both my peer groups and anyone that comes in contact with me even right now being you." (4)*
	Broad spectrum of infection transmission	*"In fact, they're airborne, they're waterborne, through direct contact, there is a broad spectrum, I guess." (2)*<br>*"Infections are very easily communicable. It's transfused very easily. One person to another person, through sneezing or coughing." [….] "In hospital, we are very prone to infections like there are lots of patients. So, if we do not use masks, we can easily transmit it." (3)*<br>*[…] "It can be airborne, or it could be through contact through Fomites or Yeah, infected particles." (6)*
	High prevalence of infection	*"Well, there is a high chance of having an infection, but I think a majority of it can be prevented by simple measures." (2)*<br>*"Infectious diseases are very prevalent among healthcare workers as well as the staff who are working because we are in contact with them 24/7." […] "Since the exposure is too much. So, there is a very high chance of getting infectious Diseases and most of the people in their working experience do get infectious disease. "(5)*<br>*"They are very prevalent in India, and we are not as aware as much as the prevalence is there." (9)*
**Perception about vaccine's effectiveness**	Trust in vaccines	*"Yes, 100% trust." [1]*<br>*"Yes, they're very effective. I'm a very pro-vaccination person, I think 97- 98% they are effective." (2)*<br>*Interviewer: "So, you have a good trust in vaccines." Participant: "Yes, owing to me being in this field as well as my father being in this field." (4)*<br>*"Vaccines are mostly effective and only after confirming their efficacy, they are released into the market." (6)*<br>*"We can say after the vaccination drive, the COVID rate has decreased" (8)*
	Willingness to be vaccinated	*"No, I don't have any problem. I'm very much willing and so enthusiastic about taking my vaccines" (2)*<br>*"Yeah, yeah, definitely. I'm willing and I'll try to tell all the people to get vaccinated." (3)*<br>*"I don't have any kind of hesitation while taking any kind of vaccines and all." (8)*<br>*"Ah, not every vaccine which I am hearing about. Diseases which are more prevalent or I am more exposed to being a doctor." (9)*
	Presence of an alternative system of medicine	*"Most of the rural India that we live with still believe, as a part of our own culture in beliefs and self-healing practices" […] "people have a home remedy system, you must be having it, I am having it, but other than that we need to come forward from that and get vaccinated." (5)*<br>*"Especially people who believe more in Ayurveda and all, they don't believe in science as much as those who made remedies or things, so they are not very into it." (6)*

**Concerns related to vaccination**	Side effects of vaccination	*"[…] Benefit outweighs the risk. So as a result, we can't just completely accept the notion that the vaccines are unsafe, because of a very small percentage of people have side effects." (1)*<br>*There could be mild side effects. There could be some severe side effects also, but the bigger picture is, they save me from a deadly disease. So, that's the bigger picture we need to consider. (2)*<br>*"No, I think it's approved, or has already been approved by the government. So, there are no side effects of these vaccines." (3)*<br>*"I see that a good majority of the cases are minor reactions […] we can consider as a necessary inconvenience for gaining immunity." (4)*<br>*"Side effects and complications of vaccines can occur due to various reasons on our part […] there is a vaccine which is administered. So, there can be a problem with the person who is administering it, the manufacturer who made it, or the problem with our management of not storing it properly." (5)*<br>*"No, it's expected. It has to have some effect on you, right, so it's expected." (6)*<br>*"Yeah, there are the side effects I believe. […] Like some patients had the breathing difficulty after taking the COVID vaccines." (8)*<br>*"Ah, while I was in UG, I was very much worried about that, but since I have come across so many patients who get vaccinated so, that phobia is no more there. […] Like for OPV, I had that paralysis side effect phobia" (9)*
	Complications after the vaccination	*"When I took COVID vaccine, I suffered. Minimal fever, no serious complications." (3)*<br>*"I have faced a minor fever and malaise due to vaccines I took in Saudi Arabia, small enough to be considered." (4)*<br>*"I had COVID vaccine due to which I had fever for a day, but I wasn't concerned about it because of the long-term beneficence" (5)*<br>*"This complication depends upon the individual to individual, like some people might develop the hypersensitivity reaction with certain vaccines, but not all the people we don't know." (7)*<br>*"Whenever I take the tetanus vaccine, I have a swelling in my arms" (8)*<br>*"They sometimes develop abscesses and pain with swelling. But that's normal" (9)*
**Additional personal factors affecting vaccination**	Forgetfulness	*"The only thing that's slightly concerning is how often we need to get vaccinated for certain things and sometimes I can't keep track of them." (6)*<br>*"Yes, especially keeping track with what we have already been vaccinated for. So, considering those, we need to make sure we are taking the ones we haven't." (6)*<br>*"I think memory plays a part because I don't remember I have to get vaccinated for certain things I don't have the brain space to process all of those things." (6)*
	Previous experience	*"Because of the fact that my father was a doctor, I do not perceive secondary experiences as a direct limitation to my own decision." (4)*<br>*"Fever, mainly just fever. However, when I was vaccinated with tetanus, I had a lot of pain in my arm." (6)*<br>*"[…] So basically, it was a pleasant experience, getting vaccinated." (9)*
	Time	*"For me, factors that can affect, timing is one thing." (1)*<br>*"As a healthcare worker, I think the timing and schedule aspects should be there. People should be given leaves on their scheduled date so that they can go and get vaccinated." (2)*<br>*"We don't really get time as students and when you start working in the hospital, you rarely get time" (5)*<br>*"In the professional community, I think it's mainly lack of time […] (6)*<br>*"No, my duty is very flexible, so I can go for that." (8)*
	Phobia	*"[…] I've seen people not getting vaccination just because of needle phobia". (2)*

		“I work in a hospital, so I don’t have any type of needle phobia.” (3)<br>*“Might be phobias like needle prick” (9)*
	Strong motivation as a facilitator	*“I think affordability is an issue in India right now, but for me, as if you ask me personally if I can spend some 2000 bucks or 3000 bucks on a shoe, I will rather spend it on a vaccine.” (2)*<br>*“Some vaccines are very costly, but we compare them with life, so better to take the vaccine.” (7)*<br>*“[…] Interviewer: If you have to pay for it, would you like to go? Participant: Yeah, of course. I would like to go because that is related to my health, and I believe I have to” (8)*
**Vaccine’s affordability and accessibility**	Hard to manage expenses	*“Right in my current economic climate, I guess it would be affordable to a great extent. However, I don’t think a large majority would agree to that.” (1)*<br>*“I also want to get an HPV, but I have to take out a separate amount from my monthly allowance to get an HPV vaccine.” (5)*<br>*“Affordability is a main issue, to the person if the target population that you want to vaccinate can’t afford the treatment, then there is no point of treatment or the vaccination.” (5)*
	Cost	*“That certain vaccine which I think is quite exorbitant.” (1)*<br>*“[…] there is a continuous ad of HPV going on. […] since awareness of it is being generated […] they want to get a vaccine for it, but the government is not giving it at the price that the person can get it.” (5)*<br>*“As a person of my financial setting, I can afford these vaccines.” (4)*<br>*“I feel being a medical person, we should be given some concession or something which we are not usually given if we are going to take some vaccines” (9)*<br>*“The cost should be minimal so people can easily get it.” (3)*
	Availability of vaccine	*“I’ve never felt that has ever run out.” (1)*<br>*“Being a healthcare professional, I think they are very easily accessible, even if not in your hospital. I can get it from another hospital and being a healthcare worker, they would give me the preference of it.” (2)*<br>*“Yeah, in my hospital, easily accessible.” (3)*<br>*“Interviewer: all the vaccines are available here at your hospital? Participant: No, no. (7)*<br>*“They are easily available like those which are recommended by NIS. They are easily accessible, but other vaccines like influenza or the recent ones, they are not easily accessible to the population.” (9)*
**Role of officials and official bodies in promoting vaccination**	Enforcement	*“Yeah, I think it needs to be enforced on health care workers because I’m pro-vaccination all the way. […] Personal autonomy, that’s there, but this is for the greater good.” (1)*<br>*“Healthcare workers have to get it like a license for practice. It should be Compulsory.” (2)*<br>*“You should enforce it I think the majority of people are careless, it’s not about the fact that they do want to get vaccinated, It’s about laziness.” (2)*<br>*“No, actually not forced vaccination, but we have to make aware all the staff like you should get vaccinated. […] Not forcefully, but we have to make aware all the staff to get vaccinated. (3)*<br>*“No enforcement will be very strong.” (4)*<br>*“If the diseases are as such that it can harm in the future, then it should be enforced.” (5)*<br>*“It should be enforced preferably, but can you really question a healthcare worker saying you can’t work here just because you’re not vaccinated.” (6)*
	communication	*“I think these medical professionals already know the relevance, so how much you will communicate […] For the mass, yes, you could try a couple of times, but other than that. I don’t think. (6)*

		"Actually, communication is a very important thing […] when there are lots of the things about the vaccine, […] If we are talking with the doctor, we will know about the vaccine which is good for us." (8)<br>*"If the health care workers get to talk about the vaccine's advantages and disadvantages, the workers will think and things will be clear." (8)*
	Presence of official circulars	*"There should be a Kind of official recommendation like every person will get these vaccines if they are working here and they should have the documents" (7)*<br>*"If the hospital HR department will give the advertisement […] I will read, I'd be able to read about that then I'll Move for that." (8)*
	Strictness by the hospital in recruiting healthcare workers	*"I think they should be really strict" (1)*<br>*"Yeah, I strongly believe that actually, I think there should be a mandatory immunization course for any healthcare worker, which should be checked by the HR or something before appointing them." […] I was asked only about my Hep-B vaccination and about covid vaccination. Nothing else was asked." (2)*<br>*"Yes, strictness will help" (8)*<br>*"It should not be. […] Maybe because we don't have documents all the time." (9)*
	Freedom of choice with consent	*"Getting something injected in my body Should be freedom of my choice. But I should have it in written […] if we suffer from that particular disease, we should not get healthcare coverage of that and something else because that's our choice to suffer from that." (2)*<br><br>*"I recently underwent an elective posting for medical ethics wherein they emphasized the importance of the autonomy of the patient. So, I think, ultimately, if it's not something of the scale of a pandemic. That portion of autonomy of the patient should be preserved." (4)*<br>*"Nothing can be done without consent. […] If you can refuse to treat the people, then they can also refuse to get treated right." (5)*
**Additional system related factors affecting vaccination**	Perks	*"Nothing else should be given, especially leave. I don't think it is necessary." (1)*<br>*"Perk should be given; I think Community education is very essential" (2)*<br>*"Perks, I think, It's a double-edged sword perks and incentives. […] I will not be in support of perks because ultimately, it's a health problem and the most someone has to gain with regards to a health measure is the beneficiary itself." (4)*<br>*"Why not? I say, why not? From my perspective […] if they give, it's a big deal." (5)*<br>*"Maybe they could be given a day off at best to recover from vaccine side effects, but not more than that" (6)*<br>*"Like as a student, I can't go for my checkups or my tests because I have classes during the day and the hospitals are closed at night. I think there could be some work done from the administration side to make sure that anyone who wishes to get vaccinated gets a Free day off or gets the vaccine delivered to them." (6)*
	Cost free	*"It should be free of cost for everyone, including healthcare workers" (1)*<br>*"If you're talking about healthcare people, most of them can afford it, but it's their will not to get vaccinated. So, I think free of cost might help in a community perspective, not a healthcare perspective." (2)*<br>*"We are serving the community so for us the vaccines would be free of cost." (7)*<br>*"We don't value things which are easily accessible." (9)*
	Role of media	*"There can be a positive role when the media mentions the number of vaccinations that happen every year and there are people who have a fear of missing out, so they will take it right. The negative can also be there. There are certain sensational news outlets. They just want TRP, and they really give voice to people who don't need it or even certain misconceptions on vaccination." (1)*

Attachment 1 to: *Ahmed M, Manchana V. Vaccination status, awareness, and its correlates among healthcare workers in the Delhi-National Capital Region (NCR): a mixed-method study. GMS Hyg Infect Control. 2024;19:Doc39. DOI: 10.3205/dgkh000494*

		"There is a very big role these days in particular that it's influencing the youth […] but there is half knowledge, there is not complete knowledge, […] when it is being promoted in the social media, things must be clear. No one should use vague terms." (5)<br>*"Media is the most important thing in terms of vaccination. If the Media says, definitely every person will take the vaccine" […] This is also a double-edged sword like, people get confused (7)*
	Convenience of location	*"In nearby, all the hospitals or the dispensaries should have all the vaccines. So, in rural areas and urban areas, they both can have the vaccines." (3)*
	Myths and misinformation	*"There are some myths we have suffered so much. I think passive immunity is so strong, in our mind, that we don't want to get an active immunity. We believe in Community immunity kind of a thing, herd immunity (2)*<br>*"[…] misinformation being spread, I think that's the major problem today in the urban setting that I'm currently living." (4)*<br>*"Cultural barrier, economic barrier […] change of perception is very important" (5)*<br>*"As younger generation myths have to be broken down, they are also producing their own myths right now since they are learning half, they are also producing." (5)*<br>*"Actually, we healthcare workers feel that they are very strong. They are exposed to the antigens repeatedly, so their immunity has already been hyped" (9)*
	Awareness promotion	*"Perhaps we could carry out more camps for these." (4)*<br>*"We should tell people about the diseases. Flip side of the diseases i.e., complications." (9)*<br>*"I'm not aware about the option of vaccines available for me, so they should be taught, maybe in the hospital" (9)*

Note: Participants were allotted numbers based on the interview sequence.
